# Effects of 14 days of bed rest and following physical training on metabolic cost, mechanical work, and efficiency during walking in older and young healthy males

**DOI:** 10.1371/journal.pone.0194291

**Published:** 2018-03-12

**Authors:** Mirco Floreani, Enrico Rejc, Paolo Taboga, Alessandro Ganzini, Rado Pišot, Bostjan Šimunič, Gianni Biolo, Carlo Reggiani, Angelina Passaro, Marco Narici, Joern Rittweger, Pietro Enrico di Prampero, Stefano Lazzer

**Affiliations:** 1 Department of Medical Area, University of Udine, Udine, Italy; 2 School of Sport Sciences, University of Udine, Udine, Italy; 3 Kentucky Spinal Cord Injury Research Center, University of Louisville, Louisville, KY, United States of America; 4 Department of Kinesiology and Health Science, California State University, Sacramento, CA, United States of America; 5 Institute for Kinesiology Research, Science and Research Centre Koper, Koper, Slovenia; 6 Department of Medical, Surgical and Health Sciences, Division of Internal Medicine, University of Trieste, Trieste, Italy; 7 Department of Biomedical Sciences, University of Padova, Padova, Italy; 8 Department of Medical Sciences, Section of Internal and Cardiorespiratory Medicine, University of Ferrara, Ferrara, Italy; 9 MRC/ARUK Centre for Musculoskeletal Ageing Research, University of Nottingham, Derby Royal Hospital, Derby, United Kingdom; 10 Institute of Aerospace Medicine, German Aerospace Center (DLR), Cologne, Germany; 11 Department of Pediatrics and Adolescent Medicine, University of Cologne, Cologne, Germany; Universita degli Studi di Verona, ITALY

## Abstract

In this study, we investigated: i) the effects of bed rest and a subsequent physical training program on metabolic cost (Cw), mechanical work and efficiency during walking in older and young men; ii) the mechanisms underlying the higher Cw observed in older than young men.Twenty-three healthy male subjects (N = 16 older adults, age 59.6±3.4 years; N = 7 young, age: 23.1±2.9 years) participated in this study. The subjects underwent 14 days of bed rest followed by two weeks of physical training (6 sessions). Cw, mechanical work, efficiency, and co-contraction time of proximal muscles (vastus lateralis and biceps femoris) and distal muscles (gastrocnemius medialis and tibialis anterior) were measured during walking at 0.83, 1.11, 1.39, 1.67 m·s^-1^ before bed rest (pre-BR), after bed rest (post-BR) and after physical training (post-PT).No effects of bed rest and physical training were observed on the analysed parameters in either group. Older men showed higher Cw and lower efficiency at each speed (average +25.1 and -20.5%, P<0.001, respectively) compared to young. Co-contraction time of proximal and distal muscles were higher in older than in young men across the different walking speeds (average +30.0 and +110.3%, P<0.05, respectively).The lack of bed rest and physical training effects on the parameters analyzed in this study may be explained by the healthy status of both young and older men, which could have mitigated the effects of these interventions on walking motor function. On the other hand, the fact that older adults showed greater Cw, overall higher co-contraction time of antagonist lower limb muscles, and lower efficiency compared to the young cohort throughout a wide range of walking speed may suggest that older adults sacrificed economy of walking to improve stability.

## Introduction

Walking is generally defined as an automatic process that is predominantly controlled by subcortical structures, and normally requires conscious control only in case of a challenging environment or perturbation [1]. Walking can be characterized by its efficiency, which is defined as the ratio between mechanical work performed by muscles and metabolic cost of walking (Cw) [2]. The metabolic cost of walking (Cw), expressed as the amount of energy spent above resting to transport 1 kg body mass over a distance of 1 m, is determined by several factors such as: generating force to support body weight, performing work to redirect and accelerate the centre of mass from step to step, swinging the limbs and maintaining stability [3].

Ageing is characterized by the developing of sarcopenia [4], which is directly responsible for loss of muscle strength, functional impairment, increased risk of falling, loss of autonomy, physical disability and poor quality of life [5–7]. In particular, loss of muscle strength in elderly people has detrimental effects on walking pattern, reducing freely chosen speed, step length and swing phase compared to young people [8–13]. Furthermore, elderly adults show impaired balance and proprioceptive abilities and reduced joint range of motion [14–16]. Although no consistent findings related to the effects of these neuromuscular alterations on the mechanical work requirements of walking in older adults have been shown [17], the interest on this topic is still vivid as reported by a recent review of Aboutorabi and colleagues [18]. Indeed, these authors have commented on a series of studies that found several differences in gait parameters between old and young individuals. In particular, it emerged that muscle weakness and lower extremity strength loss, due to sarcopenia, might influence muscle activation, stiffness and power at the level of single joints. Increased hip flexor power for compensating the reduced plantar flexor power might be an example of these sarcopenia-induced neuromuscular adaptation [11]. Hence, complex motor control mechanisms that may be adopted by older people might also alter the trajectory of their body centre of mass in walking, so leading to potential changes in their mechanical work requirements. This could be one of the reasons why Aboutorabi and colleagues stated that future studies oriented on analysing the differences in gait parameters between old and young individuals should be focused on investigating the effects of aging on the centre of mass displacement in elderly subjects [18]. Ageing-related neuromuscular adaptations may also lead to simultaneous greater activity of agonist and antagonist leg muscles (co-contraction) in the gait phases [17, 19]. These negative adaptations may play a role in determining the lower efficiency and higher metabolic cost of walking observed in older individuals [17, 20].

Disuse (i.e. bed rest) further increases the detrimental effects of ageing on metabolism and muscle protein turnover [21–24]. It is also well known that disuse induces skeletal muscle atrophy with consequent loss of force production [25]. In particular, postural muscles (i.e. knee extensors and ankle plantar flexors) are more susceptible to atrophy than non-postural ones in response to disuse and unloading [26–28]. The duration of disuse plays an important role in determining the amount of muscle atrophy: in fact, shorter bed rest (7 days) induced a relatively low decrease in thigh muscle volume (about 3%) [29], while longer bed rest (20 days) promoted a 12% and 10% decrement in plantar flexor and knee extensor muscle volumes, respectively [30]. Along this line, a 29% and a 18% decreases in triceps surae and quadriceps muscles volume, respectively, were found after 89 days of bed rest [31].

Physical inactivity or bed rest during hospitalization has been proposed as a primary factor contributing to the functional decline in elderly hospitalized patients [22, 32]. Decline in muscle force related to unloading condition may have negative effects on gait descriptors and walking motor control [21], leading to a decrease in the walking economy. Importantly, lower walking economy after disuse can further contribute to the reduced daily activity in the older population [33], leading to an inactive lifestyle and its deleterious adaptations in an already frail population.

An effective intervention for mitigating ageing-induced impairment of muscle function and physical functioning is represented by physical training. For example, isotonic and isoinertial resistance training improved lower limb muscle strength as well as functional balance during standing [34–36]. However, training interventions that did not require specific resistance training equipment were also found to be effective for improving physical functioning (i.e. stair climbing) [24, 37, 38].

Then, the main purpose of the present study was to investigate the effects of a 14-day bed rest and a subsequent 2-week physical training on Cw, mechanical work, and efficiency during walking in older and young healthy subjects. We hypothesized that the neuromuscular deconditioning induced by bed rest would have negatively influenced Cw, efficiency and mechanical work, and that these negative effects would have been greater in the older subjects. We also hypothesized that physical training performed afterward would have been sufficient for the reconditioning of these parameters. The secondary aim of this study was to investigate the difference in walking pattern between young and older adults (i.e. 55 to 64 years), as most of the studies examining the causes of ageing-induced increase in Cw are based on data collected from elderly individuals (i.e. > 65 years). We hypothesized that the higher Cw observed in older adults would coincide with higher mechanical work and higher co-contraction of representative thigh and leg antagonist muscles.

## Materials and methods

### Subjects

Sixteen older adult males (Older) and seven young males (Young) participated in this study (Table 1). All subjects had a full medical history and physical examination, with the routine haematology and biochemistry screens. None of the subjects experienced any significant disease and none was taking medications regularly or made use of any medication known to influence physical performance. The study was approved by the Slovenian National Committee for Medical Ethics at the Ministry of Health (Republic of Slovenia) and conformed to the Declaration of Helsinki. The purpose and objectives were carefully explained to the subjects and written informed consent was obtained from all of them.

**Table 1:** Physical characteristics of older (N = 16) and young (N = 7) subjects before bed rest (Pre-BR), after bed rest (Post-BR), and after 2 weeks of physical training (Post-PT).

	Older (n = 16)			Young (n = 7)					
	Pre-BR	Post-BR	Post-PT	Pre-BR	Post-BR	Post-PT	G	T	G x T
Age (y)	59.6 ± 3.4			23.1 ± 2.9			0.001		
Stature (m)	1.73 ± 0.05			1.77 ± 0.07			0.192		
Body mass (kg)	79.9 ± 12.3	77.5 ± 11.7	79.3 ± 11.6	74.8 ± 8.8	71.6 ± 8.3	74.4 ± 8.1	0.310	0.001	0.352
Body mass index (kg·m^-2^)	26.6 ±4.4	25.8 ± 4.1	26.5 ± 4.2	24.0 ± 2.4	22.9 ± 2.1	23.8 ± 2.2	0.142	0.018	0.284

Results are in mean ± SD. Significance by GLM of the main effects of Group (Older *vs* Young), Time (Pre-BR *vs* Post-BR *vs* Post-PT) and Group x Time interaction (G x T).

### Study protocol

The experimental bed rest campaign was conducted at the Valdoltra Orthopaedic Hospital, Ankaran (Slovenia). The subjects spent 19 consecutive days at the hospital, including 14 days of bed rest [24]. During the 14-day bed rest, eight randomly selected older adults underwent daily 45 minutes of computerized cognitive training by navigating through virtual mazes with the use of a joystick and computer. The same eight older subjects also received a nutritional support based on 0.4 g whey protein/kg body weight/day at breakfast during the first 14 days of rehabilitation period. Since no significant effects of both cognitive and nutritional interventions were observed on reported parameters [39], the two older groups were pooled for statistical analysis [24]. During the whole bed rest procedure, constant surveillance and 24-hour medical care was provided and all subjects received an individually controlled normo-caloric diet [40] and passive physical therapy to avoid cardiovascular disorders. Subjects performed all daily activities in bed, were allowed to freely communicate, watch television and listen to the radio, read, use computer and to receive visitors. After bed rest, subjects underwent physical training, which was conducted in the same facility.

Full testing sessions were conducted one day before the beginning of bed rest (pre-BR) and the day after the end of the 14-day bed rest (post-BR). Each of them consisted in two parts: the first one concerned the assessment of anthropometric characteristics and body composition, while the second one focused on the evaluation of metabolic cost (Cw), mechanical work and muscular activation during walking at different speeds. Subjects walked constantly on a motor-driven treadmill (Zebris Medical GmbH, Isny, Germany) at the following speeds: 0.83, 1.11, 1.39, and 1.67 m·s^-1^. Each speed was maintained for 4 minutes, and there was no recovery between walking speed trials. All subjects were familiarized with walking on treadmill 2 days before the beginning of bed rest. They experienced walking at all speeds that were subsequently tested. The familiarization duration lasted about 15–20 minutes [17]. Subjects returned to the laboratory 2 weeks after the end of bed rest in order to perform the same testing sessions. During these 2 weeks, subjects underwent the physical training program reported here below.

### Physical training program

Subjects began a 28-day physical training program the second day after the end of BR; however, only the initial 14 days of physical training were considered in this study, i.e the same duration of the disuse period. Physical training consisted of 6 sessions in total (3sessions/week); each session, which lasted about 65 minutes, was followed by 1 or 2 days of routine daily activity. Physical training was aimed at reconditioning both the neuromuscular and aerobic systems, proposing a series of exercises that did not require specific training equipment so that they could be translated to any home and community environment. The first 12 minutes of each training session were devoted to warm-up; subjects performed 6 minutes of Nordic walking, its speed being determined from a 2-km walking test performed before BR, and 6 minutes of active stretching (10 exercises). Then, subjects performed 20 minutes of strength and balance exercises. This section started with half squat (1 set; 10 repetitions; overload: from no overload to a 6 kg ball held with both hands), and continued with a circuit training (30 seconds of exercise followed by 30 seconds of rest) comprised of 8 motor tasks. In particular, the following strength exercises focused on lower limbs were proposed: frontal and sagittal plane lunge, double leg heel raise with elastic resistance; hip extension with elastic resistance. Also, balance exercises mainly consisted of dynamic standing balance activities (i.e. standing on toes; standing on balance foams) and functional movements that involved reaching and passing objects. Strength and balance exercises were followed by 30 minutes of aerobic exercise (e.g. Nordic walking; brisk walking, running). The last 3 minutes were devoted to cool down (relaxation and breathing exercises). Subjects’ heart rate was preventively monitored during each training session. Training was conducted at the hospital and in the gym near the hospital and supervised by 6 physical trainers who instructed the subjects to properly perform the different exercises. All subjects performed all planned training sessions.

### Anthropometric characteristics

Body mass was measured to the nearest 0.1 kg with a manual weighing scale (Seca 709, Hamburg, Germany) with the subject dressed only in light underwear and without shoes. Stature was measured to the nearest 0.5 cm on a standardized wall-mounted height board.

### Metabolic cost of walking

In each testing session, measurements of resting oxygen consumption (V’O_2_), carbon dioxide (V’CO_2_) production and heart rate (HR) were carried out by using a metabolic unit (Quark-b^2^, Cosmed, Italy). Ventilatory and gas exchange responses were measured continuously. The volume and gas analysers were calibrated using a 3-liter calibration syringe and standard calibration gases (16.00% O_2_; 4.00% CO_2_). Each participant stood quietly relaxed for five minutes whilst metabolic measures were being collected breath by breath. Real-time plots of V’O_2_, heart rate, and respiratory exchange ratio were closely monitored during the last minute of each walking speed to ensure that metabolic steady state was reached. Data post processing included the calculation of mean values of V’O_2_, V’CO_2_ and HR over the fourth (and last) minute of each walking speed, which were considered for further analysis.

In addition, respiratory exchange ratio (RER) was monitored to ensure that it remained under the specific threshold of 1.0. All these precautions were required to indicate that metabolism was essentially oxidative. The metabolic cost of walking (C_W_, J·(kg·m)^-1^) was calculated by dividing net energy expenditure (obtained by subtracting pre-exercise standing V’O_2_ from gross V’O_2_ and converted to joules according to the formula given by Garby and Astrup [41], which accounts for the RER-dependent variation of O_2_ energy equivalence) by speed and body mass.

### Mechanical work

Three-dimensional kinematic information was collected using two digital cameras (Basler—Pilot, Ahrensburg, Germany) sampling at 210Hz [42–44]. The cameras were positioned symmetrically 5 m behind the treadmill, spaced about 6 m one from the other with an angle between the respective optical axes of about 65°. Two cubic 1x1x1m metal boxes with markers at every edge were used for calibration purposes and for the setting of a laboratory frame of reference. The position of every marker was precisely measured so that the measurement error was less than 0.5 mm. The calibration plane was placed between the cameras locations and the subject so that it resulted precisely within the calibrated volume throughout the walking tests. A calibration video was recorded. Digitalization of the images from this video provided us with the coefficients required to perform the direct linear transformation (DLT) technique included in the video analysis software (see below). DLT technique has been proven to lead to very good results in reconstructing the three-dimensional data from photographic observations of either objects or human bodies engaged in locomotion [45–47]. Kinematic analysis of the following body segments was performed: head-trunk (ear lobe, anterior to tragus of ear-iliac crest), thigh (great trochanter-lateral epicondyle of femur), shank (lateral epicondyle of femur- lateral malleolus), foot (calcaneus-5^th^ metatarsal head), upper arm (shoulder-elbow), and forearm (elbow-wrist) [17, 48]. Reflective markers (0.75 cm radius plastic ball) that contrasted the environmental lights and colors were positioned on the body reference points mentioned above. A 3D software (SIMI motion 3D) was used to track and reconstruct the three-dimensional position of each marker. The tracked data were filtered through a moving-average filter (radius = 1) [49]. Appropriate regression equations [50] that considered anthropometric data of the 11 rigid segments (head-trunk, upper arms, forearms, thighs, shanks and feet) were then used to compute the position of the segments, the body center of mass (COM_wb_), and mechanical work. This analysis was performed by using a custom-made Matlab routine (v6.3, Mathworks, Inc, USA).

Total mechanical work (W_TOT_) performed during walking is described as the sum of two separate entities: the mechanical external work (W_EXT_) and the mechanical internal work (W_INT_) [2, 51]. W_EXT_ represents the work done to raise and accelerate the COM_wb_ within the environment [51], whilst W_INT_ is the work necessary to accelerate the limbs with respect to the COM_wb_ [2, 51]. We calculated W_INT_ as the sum of the positive increments in the kinetic energy of each limb according to the procedure described by Mian and colleagues [17]. W_EXT_ was calculated as the sum of positive increments in the total mechanical energy of COM_wb_ in agreement with the procedure described by Mian and colleagues [17], which was already used also by our research group [49]. W_TOT_, W_INT_ and W_EXT_ were obtained from ten consecutive representative strides (i.e. without anomalous movements of limbs, torsion of the head or trunk, etc.). We calculated the interchange of the mechanical energies of the centre of mass (pendulum-like mechanism) using the recovery index (R) as adopted by Cavagna and colleagues [52] and presented in the later work of Mian’s group [17]. We determined the efficiency (EFF) of the total positive work produced during walking of each subject dividing W_TOT_ by C_W._ We determined also the walking stride frequency (SF, expressed as strides·min^-1^) by calculating the total number of peak vertical displacements of a marker positioned at the level of the calcaneus in the right foot, over each minute of walking.

### Surface electromyography recordings

Surface electromyography (EMG) was collected from four muscles of the right lower limb: *vastus lateralis* (VL), *biceps femoris* (BF), *gastrocnemius medialis* (GM) and *tibialis anterior* (TA). Pre-gelled surface EMG electrodes (circular contact area of 1 cm diameter, BIOPAC Systems, Inc., USA) were placed (inter electrode distance equal to 20 mm) at the following locations [53]: a) for VL at two-third on the line from the anterior superior iliac spine to the lateral side of the patella; b) for BF midway between the ischial tuberosity and the lateral epicondyle of the tibia; c) for GM on the most prominent bulge of the muscle; d) for TA at one third on the line between the tip of the fibula and the tip of the medial malleolus. In order to ensure a good electrode-skin interface, prior to the application of the electrodes, the subject’s skin was shaved, rubbed with an abrasive paste, cleaned with an alcohol solution, and dry-cleaned with gauze. EMG data were sampled at a frequency of 2 kHz, and recorded by a four-channel electromyography system (EMG100C, BIOPAC Systems, Inc., USA; Band-pass Filter: 10–500 Hz; RMS Noise Voltage: 0.2 μV; Input impedance: 2 MΩ; Common Mode Rejection Ratio: 110 dB). In order to place electrodes in the same anatomical location during the three different experimental sessions, electrodes position was marked on acetate paper using moles and small angiomas (which may be assumed to maintain a fixed position) as reference points. The EMG electrodes were fixed at the beginning of each experimental session and were not removed between walking tests.

EMG raw signal recorded between the third and fourth minute of each walking bout performed by the subject was full-wave rectified and then low-pass filtered with a cut-off frequency of 10 Hz. The determination of EMG onset and offset activity of each muscle was achieved by using a computer-automated procedure [17]. Visual inspection of EMG activity was added in order to monitor the suitability of the algorithm used. In particular, we calculated the amount of stride duration (% of stride duration) in which two representative antagonist muscles were active at the same time (co-contracted). Eight representative strides for each walking speed were considered to calculate the average co-contraction value for both proximal, thigh muscles (VL and BF) and distal, leg muscles (GM and TA).

### Statistical analysis

Statistical analyses were performed using PASW Statistic 18 (SPSS Inc., IL, USA) with significance set at P<0.05. All results are expressed as means ± SD. Normal distribution of the data was tested using the Shapiro-Wilk test. Sphericity (homogeneity of covariance) was verified by the Mauchly’s test. When the assumption of sphericity was not met, the significance of the F-ratios was adjusted according to the Greenhouse-Geisser procedure.

Differences in anthropometric characteristics and body composition of older and young subjects, before (pre-BR) at the end (post-BR) and 14 days after physical training (post-PT), were studied with General Linear Model repeated measures with two factors considering ANOVA of the main effects of group (G: Older *vs* Young), time (T: pre-BR *vs* post-BR *vs* post-PT) and group x time interaction.

Changes of Cw, mechanical and electromyographic recordings, were studied with General Linear Model repeated measures with three factors considering groups (G: Older *vs* Young), time (T: pre-BR *vs* post-BR *vs* post-PT) and speeds (considering four different speeds, S: 0.83, 1.11, 1.39, 1.67 m·s^-1^). When no difference was found across time on Cw, mechanical and electromyographic outcomes, the values of pre-BR, post-BR and post-PT were averaged and compared between groups (G: Older *vs* Young) as a function of speeds (S: 0.83, 1.11, 1.39, 1.67 m·s^-1^).

Multiple comparisons were made with the Tukey HSD post hoc test when the Greenhouse-Geisser epsilon correction factor was P > 0.50 or with the Bonferroni post hoc test when the epsilon was P < 0.05.

## Results

### Physical characteristics of subjects

Baseline values of stature, body mass and body mass index (BMI) were not significantly different between groups at baseline (Table 1). Bed rest induced significant body mass decrease (P<0.001) in Older (-3.1%) and Young (-4.4%). Physical training performed after bed rest increased body mass in both groups (+2.4% and +3.9%, P<0.001, in Older and Young, respectively).

### Metabolic measurements

**Fig 1:**
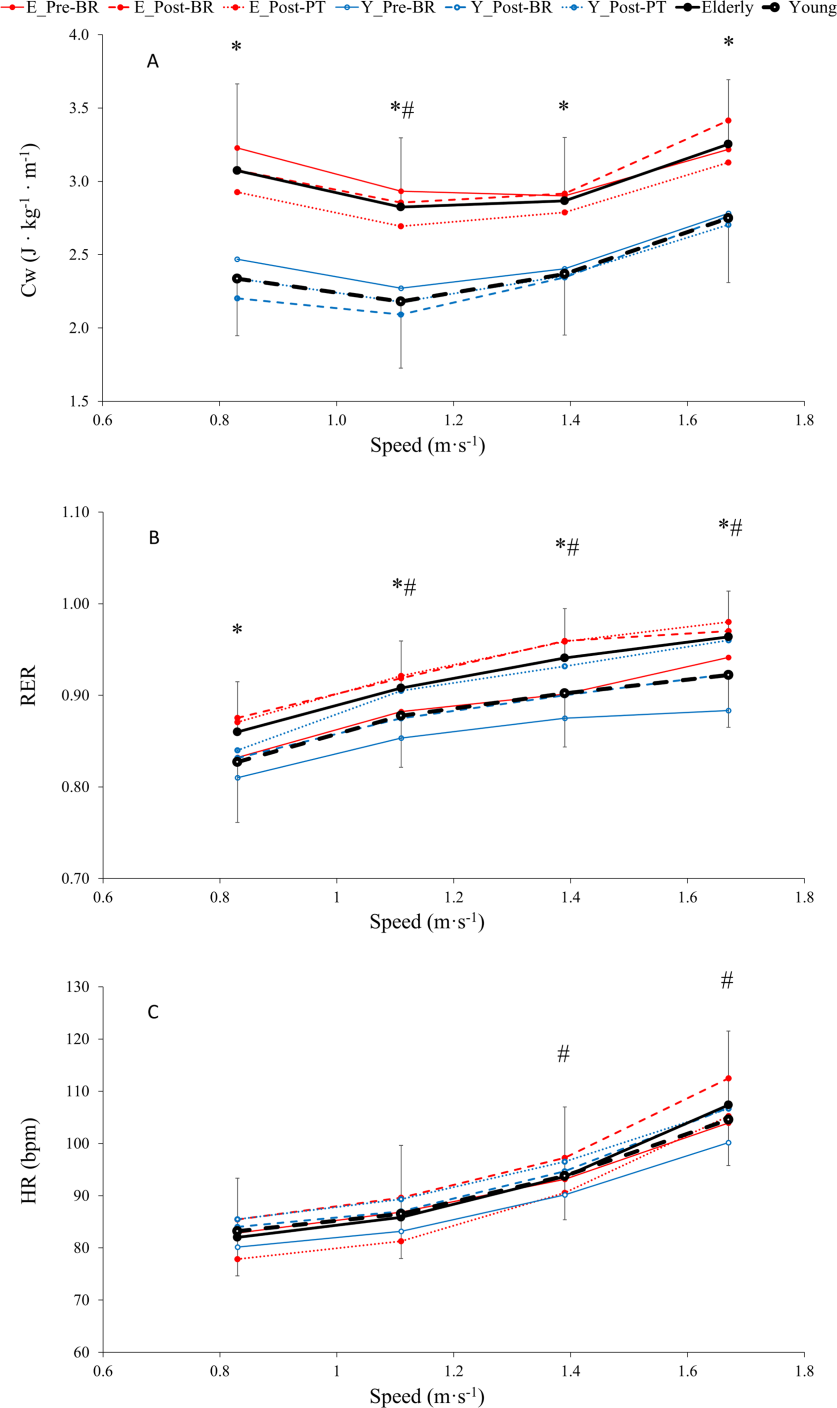
**Averaged values of metabolic cost of walking (Cw, A), respiratory exchange ratio (RER, B) and heart rate (HR, C) across time (pre-BR, post-BR and post-PT) as a function of speed, in older (-●-) and young (- o -) subjects (see statistics paragraph).** *(The lines represent mean values obtained before (solid line) and after (dashed line) bed rest*, *and 14 days after physical training (dotted line) in older (red full circle) and young (blue open circle) subjects)*. Results are in mean ± SD. *: P < 0.001, Older group is significantly different than Young at a given speed. #: P < 0.001, values at given speeds are significantly different than at 0.83 m · s^-1^. GLM results reported in main text.

No effects of bed rest and physical training were observed on metabolic cost of walking (Cw), respiratory exchange ratio (RER) and heart rate (HR) in both groups (Fig 1, Table 2).

**Table 2:** Metabolic, mechanical work and electromyography recordings results of General Linear Model repeated measures with three factors considering group (G: Older vs young), time (T: Pre-BR *vs* Post-BR *vs* Post-PT), speeds (considering four different speeds, S: 0.83 *vs* 1.11 *vs* 1.39 *vs* 1.67 m·s^-1^) and interaction (G x T x S).

	Group	Time	Speeds	G x T x S
***Metabolic***				
Metabolic cost of walking	0.001	0.141	0.001	0.849
Respiratory exchange ratio	0.001	0.141	0.001	0.344
Heart rate	0.146	0.302	0.001	0.658
***Mechanical work***				
External work	0.004	0.370	0.001	0.628
Internal work	0.687	0.328	0.001	0.582
Total work	0.016	0.981	0.001	0.650
Recovery	0.017	0.836	0.001	0.977
Stride Frequency	0.042	0.911	0.001	0.123
Efficiency	0.001	0.734	0.001	0.498
***Electromyography***				
Proximal co-contraction time	0.038	0.797	0.001	0.310
Distal co-contraction time	0.043	0.707	0.001	0.238

Mean values of C_W_ were significantly higher in Older than in Young at each speed by an overall mean of 25.1% (P<0.001, Fig 1A, Table 2). Furthermore, C_W_ changed with speed in both groups and was significantly lower at 1.11 and 1.39 m∙s^-1^ than at 0.83 and 1.67 m∙s^-1^ (average: -11.5%, P<0.001, for Older; -15.5%, P<0.001, for Young).

Mean values of respiratory exchange ratio (RER) were significantly higher in Older than in Young at each speed by an overall mean of 4.0% (P<0.001, Fig 1B, Table 2) and remained lower than 1 for each speed in Older and in Young. RER increased significantly with speed in both groups. RER at 1.11, 1.39 and 1.67 m∙s^-1^ were significantly higher than RER measured at 0.83 m∙s^-1^ in Older (by 5.6, 9.4 and 12.1%, respectively, P<0.001, Table 2) and in Young (by 6.1, 9.1 and 11.5%, respectively, P<0.001, Table 2).

Mean values of heart rate (HR) were not significantly different between groups at each speed (Fig 1C, Table 2). Then, HR was significantly higher at speeds 1.39 and 1.67 m∙s^-1^ (by 14.2 and 30.9% for Older and by 12.7 and 25.6% for Young, P<0.001, Table 2) than at speed 0.83 m∙s^-1^.

### Mechanical measurements

**Fig 2:**
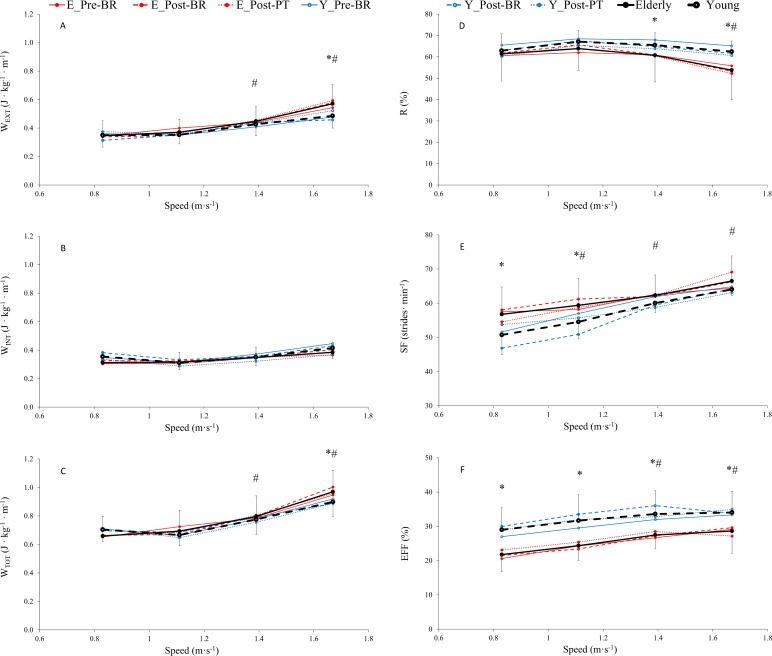
**Averaged values of external work (W**_**EXT**_**, A), internal work (W**_**INT**_**, B), total work (W**_**TOT**_**, C), recovery (R, D), stride frequency (SF, E) and efficiency (EFF, F) across time (pre-BR, post-BR and post-PT) as a function of speed, in older (-●-) and young (- o -) subjects (see statistics paragraph).** *(The lines represent mean values obtained before (solid line) and after (dashed line) bed rest*, *and 14 days after physical training (dotted line) in older (red full circle) and young (blue open circle) subjects)*. Results are in mean ± SD. *: P < 0.05, Older group is significantly different than Young at a given speed. #: P < 0.001, values at given speeds are significantly different than at 0.83 m · s^-1^. GLM results reported in main text.

No effects of bed rest and physical training were observed on total work (W_TOT_), recovery index (R), stride frequency (SF) and efficiency (EFF) (Fig 2, Table 2).

Mean values of W_EXT_ and W_TOT_ at each point were significantly higher in Older than in Young (Fig 2A and 2C, Table 2) at speed 1.67 m∙s^-1^ (by 17.5 and 7.8%, respectively, P<0.05). W_EXT_ and W_TOT_ increased significantly as a function of speed (Fig 2A and 2C, Table 2). W_EXT_ and W_TOT_ were significantly higher at speeds 1.39 m∙s^-1^ (by 29.1 and 20. 8%, respectively, for Older; and by 22.1 and 10.1%, respectively, for Young; P<0.001) and 1.67 m∙s^-1^ (by 64.9 and 47.2%, respectively, for Older; and by 39.1 and 27.6%, respectively, for Young, P<0.001) than at 0.83 m∙s^-1^. W_INT_ values were not significantly different between the two groups across all walking speeds. At speeds of 1.11, 1.39 and 1.67 m∙s^-1^, mean W_INT_ values of Older and Young tended to be greater than those observed at 0.83 m∙s^-1^ although not statistically significant.

Mean values of R were significantly lower in Older than in Young (Fig 2D, Table 2) at speeds 1.39 and 1.67 m∙s^-1^ (by 7.3 and 13.9%, respectively, P<0.05). Moreover, R decreased significantly in Older as a function of speed and was 12.4% lower at 1.67 m∙s^-1^ than at 0.83 m∙s^-1^ although not statistically significant.

Mean values of SF (Fig 2E, Table 2) were significantly higher in Older both at 0.83 and 1.11 m∙s^-1^ by 11.8 and 8.9%, respectively (P<0.05). Moreover, SF increased as a function of speed and was significantly higher at 1.11, 1.39 and 1.67 m∙s^-1^ than at 0.83 m∙s^-1^ in both groups (by 4.6, 9.7 and 17.2% in Older, P<0.05; and 7.5, 18.3 and 26.2% in Young, P<0.05).

Mean values of EFF (Fig 2F, Table 2) were significantly lower in Older among all speeds by an overall mean of -20.5% (P<0.001). Additionally, EFF increased as a function of speed and was significantly higher at 1.39 and 1.67 m∙s^-1^ than at 0.83 m∙s^-1^ in both groups (by mean 29.0% in Older and by 16.6 in Young, P<0.001).

### Electromyographic measurements

**Fig 3:**
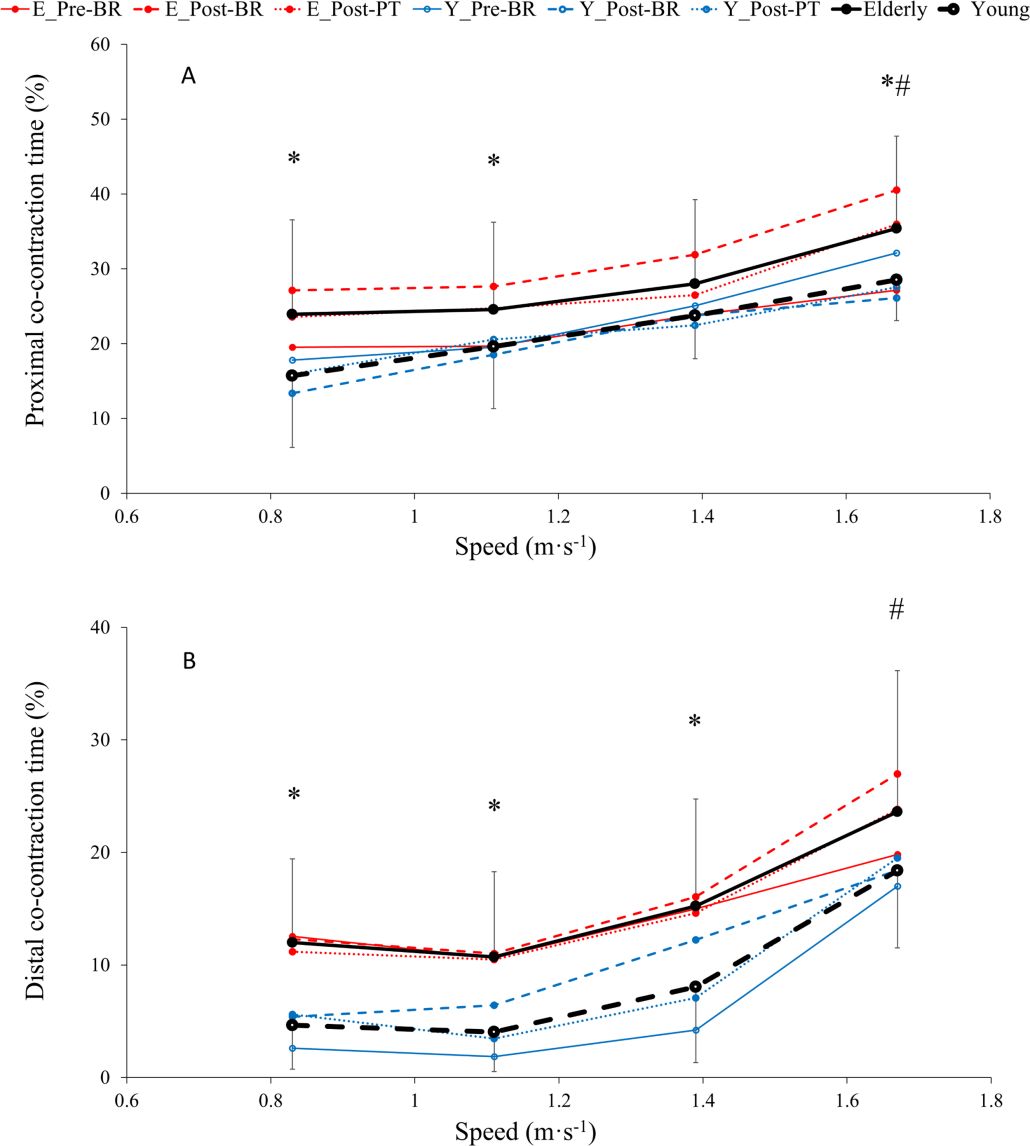
**Averaged values of proximal (*vastus lateralis* and *biceps femoris*, A) and distal (*gastrocnemius medialis* and *tibialis*, B) co-contraction time across time (pre-BR, post-BR and post-PT) as a function of speed, in older (-●-) and young (- o -) subjects (see statistics paragraph).** *(The lines represent mean values obtained before (solid line) and after (dashed line) bed rest*, *and 14 days after physical training (dotted line) in older (red full circle) and young (blue open circle) subjects)*. Results are in mean ± SD. *: P < 0.05, Older group is significantly different than Young at a given speed. #: P < 0.001, values at given speeds are significantly different than at 0.83 m · s^-1^. GLM results reported in main text.

No effects of bed rest and physical training were observed on co-contraction time of proximal and distal muscles in Older and Young (Fig 3, Tab. 2).

Mean co-contraction time values of proximal muscles (VL-BF, Fig 3A, Table 2) were significantly higher in Older than in Young at speeds 0.83, 1.11 and 1.67 m∙s^-1^ (average 52.3, 25.2 and 24.2%, respectively, P<0.05). Mean co-contraction time of distal muscles (GM-TA, Fig 3B, Table 2) were significantly higher in older than in young subjects at speeds equal to 0.83, 1.11 and 1.39 m∙s^-1^ (average 157.7, 165.7 and 89.1%, respectively, P<0.05).

Co-contraction time increased as a function of speed for proximal muscles and was significantly higher at 1.67 m∙s^-1^ compared to 0.83 m∙s^-1^ in Older as in Young (by 48.0 and 81.4%, respectively, P<0.001). Similarly, co-contraction time of distal muscles was higher at 1.67 m∙s^-1^ compared to 0.83 m∙s^-1^ (by 97.0 and 295.2% in Older and Young, respectively, P<0.001).

## Discussion

The main results of the present study showed that: 1) 14 days of bed rest and the following physical training did not influence Cw, mechanical work and co-contraction time during walking at different speeds in both older and young subjects; 2) before, after bed rest and after physical training, older subjects showed higher Cw, SF and lower R and EFF than young subjects, and 3) co-contraction time of proximal and distal muscles were higher in Older than in Young across the different walking speeds.

As reported in our previous work [24], for the same population examined in the present study, bed rest induced several undesirable consequences in both older and young subjects; also, partial recovery was observed after physical training. In particular, bed rest caused a significant reduction in total lean mass and quadriceps muscle volume as well as a significant increase in the percentage of body fat. These changes occurred in concert with decreased strength (i.e., maximal voluntary isometric force and explosive power of lower limb) and fitness level (i.e., V’O_2_peak) [24]. While all parameters describing muscle volume and function showed a complete recovery at the end of 14 days of physical training in young subjects, an incomplete recovery of quadriceps volume, explosive power of lower limbs and V’O_2_peak was observed in the older group [24].

In spite of the abovementioned adaptations brought about by bed rest and physical training, no changes in Cw, mechanical work and co-contraction time during walking were induced by these two interventions. This unexpected finding was likely related to the healthy status of older individuals, which could have mitigated the negative effects of bed rest on walking motor function. The age of older subjects (mean 60 years; range: 53–65 years) may have also played an important role, as a normal gait pattern is retained in 85% of individuals aged 60 and only in 18% of individuals aged 85 [54]. Furthermore, bed rest-induced adaptations on walking speed and functional parameters were reported in elderly subjects whose mean age was between 7 and 10 years higher than the group of older adults investigated in the present study [21, 33]. Our results can be also explained by the fact that walking is a relatively basic and automatic motor task that is primarily controlled at the spinal level with the contribution of sensory information derived from the lower limbs [55]. Walking is also an optimized human gait, as for example changes in gravity ranging between 1 g (Earth) and 0.17 g (Moon) seem to have limited effects on Cw, while the cost of transportation of other types of locomotion (i.e. running; hopping) is affected to a much greater extent by this change in gravity [56]. A previous study by our group also supports the view that bed rest-induced neuromuscular adaptations may not affect some motor patterns substantially controlled by the spinal cord, as bilateral power deficit of lower limbs and co-contraction between knee extensors and flexors assessed during explosive efforts were not altered after 35 days of bed rest in young healthy volunteers [57]. Hence, it seems plausible that, in healthy older adults, 14 days of bed rest may not be sufficient for disrupting walking pattern, even if lower limb muscle and cardiovascular performance impaired [24]. From a rehabilitative perspective, walking on flat, even surfaces seems a physical activity that can be proposed immediately after prolonged periods of disuse (i.e. 2 weeks of hospitalization) in older adults that have an otherwise healthy neuromuscular system, as mechanics and economy of walking remain similar as they were prior to disuse. This could help preventing the vicious circle often observed in older individuals who further reduce their daily physical activity after hospitalization [33].

In the present study, Cw was about 25.1% higher in older adults than in young subjects. This finding is in agreement with previous studies that observed an increased Cw related to ageing [17, 58–61]. Decreased strength, metabolic rate, and maximal oxygen consumption are some of the age-related consequences that can influence Cw. In older and elderly adults, subtle changes in the pattern and neuromuscular control of locomotion may result in altered trajectories of the body centre of mass and the limbs, and changes in posture such as increasing trunk flexion. These adaptations may lead to an increased mechanical work, thus contributing to an increase in V’O_2_ [18]. However, in the present study, Older and Young performed similar W_INT_, W_EXT_ and W_TOT_ to lift and accelerate the centre of mass during walking, even though Older showed higher Cw than Young. Moreover, only at 1.67 m·s^-1^ Older showed higher W_EXT_, W_TOT_ and lower R than Young. It is interesting to note that the literature presents a variety of W_INT_ values that tend to be either greater [2], smaller [62] or more similar [17] to those reported in the present study. Also, W_INT_ values observed in this study tend to increase as a function of walking speed, even if this trend is not significant and less marked compared to the data previously reported [2, 17, 48, 62]. The peculiar trend of data reported in the present manuscript can be due to the filtering procedure used for the 3D coordinate analysis and to the motion capture technique, which is slightly different compared to the multi-camera systems presently available in the market. These observations have been already reported in literature. In fact, Nardello and colleagues [62] concluded that the adopted filtering protocol, among all the other methodological differences, can be responsible for the W_INT_ discrepancies presented in different papers.

The differences in walking pattern between young and older individuals found in this study do not always agree with those found in other studies. For example, Mian and co-workers reported greater W_INT_ values in elderly compared to young subjects [17]. This discrepancy may be explained by the fact that age of the older group is lower in the present study compared to the cited reference (i.e. mean age = 74 ± 3 years in [18]).

However, the fact that W_INT_, W_EXT_ and W_TOT_ were overall not significantly different between the two groups of the present study suggests that other mechanical factors, such as changes in the metabolic cost of stabilizing the body, the efficacy of muscular system and/or the amount of co-contraction, may have contributed to the greater Cw observed in Older. Also, the fact that visual and vestibular functions are generally impaired with ageing [63, 64] may favour novel strategies for stabilizing the body during walking [65], and consequently increasing Cw. However, metabolically expensive strategies to improve stability such as increased co-contraction of antagonist muscles may not be detected by mechanical analyses and still contribute to the greater Cw in older individuals [17, 66–68]. In the present study, EMG recordings were helpful to detect alterations in co-contraction pattern of lower limb muscles during walking. In fact, we detected longer co-contraction times both in proximal and distal muscles in Older compared to Young. Interestingly, similar results were obtained by Mian and co-workers for the co-contraction time of thigh muscles [17]. Increased co-contraction is interpreted as compensatory mechanism to increase joint stiffness and stability, and it is associated with normal, healthy ageing [66, 69]. Despite the beneficial role that this mechanism might play in older and elderly adults to promote safer walking, it might be also disadvantageous because it can increase the cost of locomotion [17, 69, 70]. Our results are in agreement with these studies and support the view that older adults tend to adapt walking pattern increasing co-contractions of antagonist muscles to conceivably improve stability and safety, even if this leads to sacrifice walking economy. Another ageing-related factor that could contribute to the greater Cw in older subjects is the decreased efficiency of the muscle itself, which would then require more metabolic energy to perform a given amount of mechanical work [17, 71].

In conclusion, 14 days of bed rest and the following physical training did not induce any adaptation on Cw, mechanical work, efficiency and co-contraction of antagonist lower limb muscles during walking in healthy young and older individuals. In addition, older adults presented higher Cw, stride frequency, co-contraction time of proximal and distal antagonist muscles and lower efficiency compared to young subjects.
